# Association between oxidative balance score and allergic rhinitis in American adults: a cross-sectional study

**DOI:** 10.3389/fnut.2025.1655316

**Published:** 2025-09-25

**Authors:** Yanhua Tian, Wenbo Sun, Jijun Song

**Affiliations:** ^1^Department of Otorhinolaryngology, Zhoukou Central Hospital, Zhoukou Medical Science Research Center, Zhoukou, China; ^2^Department of Pharmacy, Zhoukou Central Hospital, Zhoukou Medical Science Research Center, Zhoukou, China

**Keywords:** oxidative balance score, allergic rhinitis, NHANES, diet, lifestyle

## Abstract

**Background:**

Oxidative stress has been implicated in the development of allergic rhinitis (AR), yet its population-level impact using composite oxidative metrics remains underexplored. This study aimed to investigate the association between the oxidative balance score (OBS), a composite indicator of pro- and antioxidant exposures, and AR in U.S. adults.

**Methods:**

We analyzed data in the 2005–2006 NHANES. An OBS, comprising 16 dietary and 4 lifestyle factors, was calculated and analyzed as both a continuous variable and by quartiles. Associations with AR were assessed using multivariable logistic regression and restricted cubic spline (RCS) models. Survey-weighted analyses were additionally performed to account for the complex sampling design and assess national representativeness. Sensitivity analyses included dietary energy adjustment (residual method), exclusion of asthma or supplement users, and alternative AR definitions. Exploratory subgroup analyses were further conducted across demographic and clinical strata with false discovery rate (FDR) correction for multiple testing.

**Results:**

Among 1,491 adults, higher total and dietary OBS were significantly associated with AR. In fully adjusted models, each unit increase in total OBS corresponded to 2% higher odds of AR (OR = 1.02; 95% CI: 1.01–1.04), and each SD increase to 19% higher odds (OR = 1.19; 95% CI: 1.06–1.34). Dietary OBS showed a similar association (OR = 1.03; 95% CI: 1.01–1.04), whereas lifestyle OBS was not significant (OR = 1.01; 95% CI: 0.93–1.08). Quartile analyses revealed dose–response relationships, with Q4 of total (OR = 1.67; 95% CI: 1.20–2.33) and dietary OBS (OR = 1.60; 95% CI: 1.16–2.22) showing significantly increased odds compared with Q1. In survey-weighted analyses, total OBS remained significantly associated with AR, whereas dietary and lifestyle OBS were not. These associations remained robust in sensitivity analyses.

**Conclusion:**

In a nationally representative sample, higher OBS was associated with greater odds of AR after adjustment, with an approximately linear association. Associations were stronger for dietary than lifestyle OBS, and the positive association persisted for total OBS in survey-weighted models. Overall, these findings suggest OBS may serve as a practical composite marker of diet-related redox balance in AR epidemiology.

## Introduction

Allergic rhinitis (AR) is a prevalent, nonfatal, chronic inflammatory disorder of the nasal mucosa, typically presenting with sneezing, nasal congestion, rhinorrhea, and nasal itching (1). It affects approximately 10–30% of the global population, with its prevalence rising notably in urbanized regions with high environmental exposure burdens over recent decades (2, 3). Although nonfatal, AR imposes a substantial burden on quality of life, work productivity, and healthcare resources (4). AR also frequently coexists with other atopic conditions such as asthma and atopic dermatitis and is recognized as part of the “atopic march”—a progressive sequence of allergic manifestations during early life (5). Its pathogenesis involves a complex interplay between IgE-mediated hypersensitivity and immune cell activation, typically triggered by environmental allergens (6, 7).

**Figure 1:**
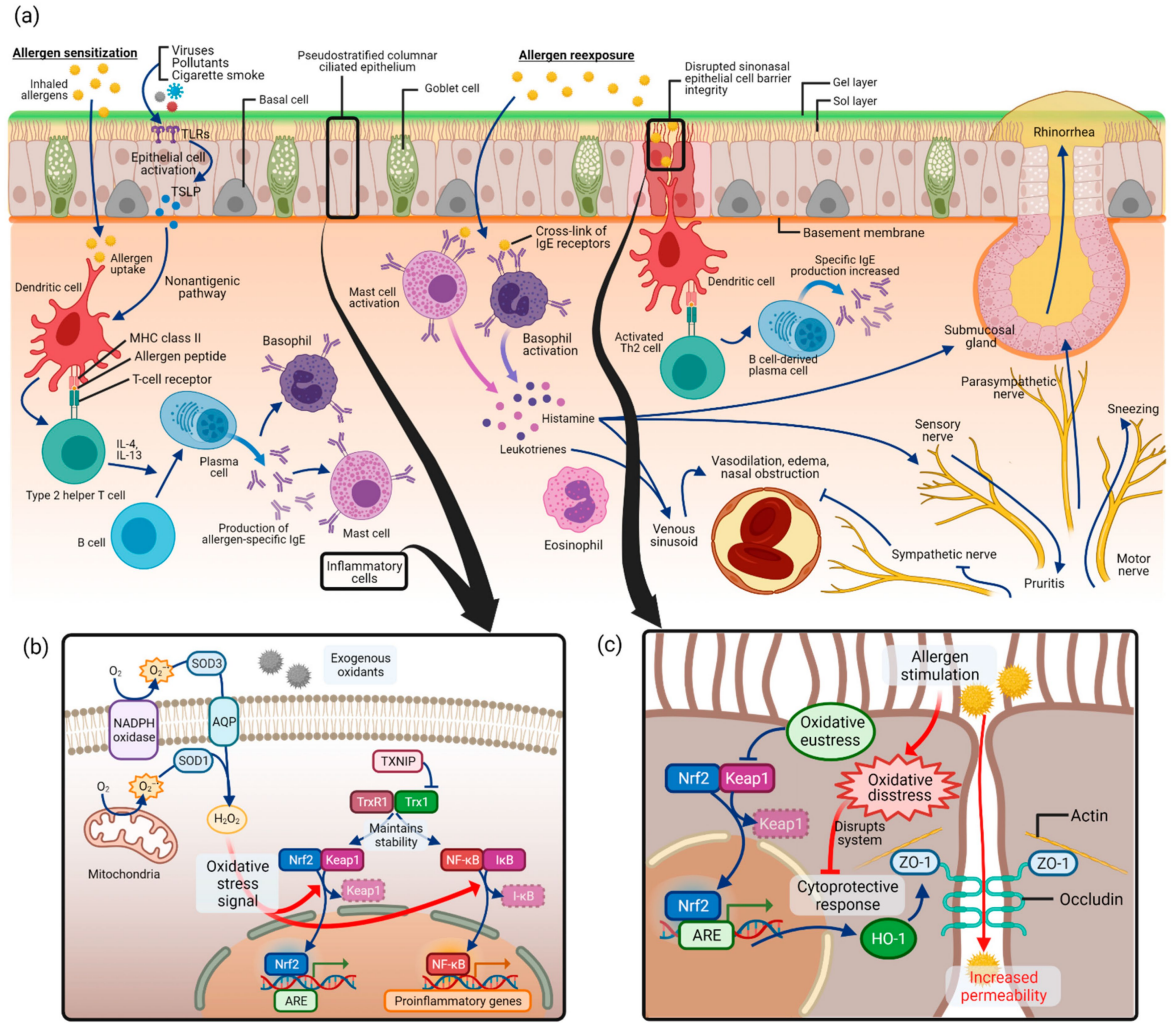
Proposed pathways linking oxidative stress and AR. Adapted from (11), licensed under CC BY 4.0. **(a)** Allergic sensitization and re-exposure trigger inflammatory responses. **(b)** Excessive reactive oxygen species (ROS: superoxide anion, O₂^−^; hydrogen peroxide, H₂O₂) activate oxidative stress signaling, particularly the NF-κB pathway, while Nrf2 mediates antioxidant defense. **(c)** Oxidative stress disrupts sinonasal epithelial barrier integrity, thereby enhancing allergen penetration and allergic inflammation. Created with BioRender.com.

Emerging evidence has highlighted the pivotal role of oxidative stress in the pathogenesis of allergic diseases, including AR (8, 9). Oxidative stress results from an imbalance between the excessive generation of reactive oxygen species (ROS) and the antioxidant defense system (10). In AR, In AR, ROS such as O₂^−^ and H₂O₂ can disrupt epithelial barrier integrity, activate redox-sensitive transcription factors such as NF-κB, and amplify the release of proinflammatory mediators, thereby enhancing allergen sensitization and perpetuating Th2-driven immune responses (8). Moreover, oxidative stress impairs epithelial tight junctions, increases mucosal permeability, and promotes mast cell and eosinophil activation, ultimately leading to nasal obstruction, rhinorrhea, and sneezing. Clinical and experimental studies consistently demonstrate that patients with AR exhibit elevated markers of oxidative stress and reduced antioxidant capacity (11). Collectively, these findings suggest that ROS are not merely byproducts of allergic inflammation but act as key drivers of epithelial dysfunction, immune dysregulation, and symptom exacerbation in AR. A schematic overview of these mechanisms is illustrated in Figure 1.

The oxidative balance score (OBS) is a composite metric that quantifies the net balance of pro-oxidant and antioxidant exposures derived from both dietary and lifestyle factors (12, 13). A higher OBS reflects a more favorable oxidative status, potentially protective against oxidative damage and inflammation. Previous studies have linked elevated OBS with reduced risk of chronic conditions such as cardiovascular disease, metabolic syndrome, and cancer (14, 15). However, limited data are available regarding the relationship between OBS and allergic diseases such as AR. Given these gaps, we aimed to investigate the association between OBS and AR in U.S. adults using data from the 2005–2006 cycle of the National Health and Nutrition Examination Survey (NHANES).

## Methods

### Data source and study population

**Figure 2:**
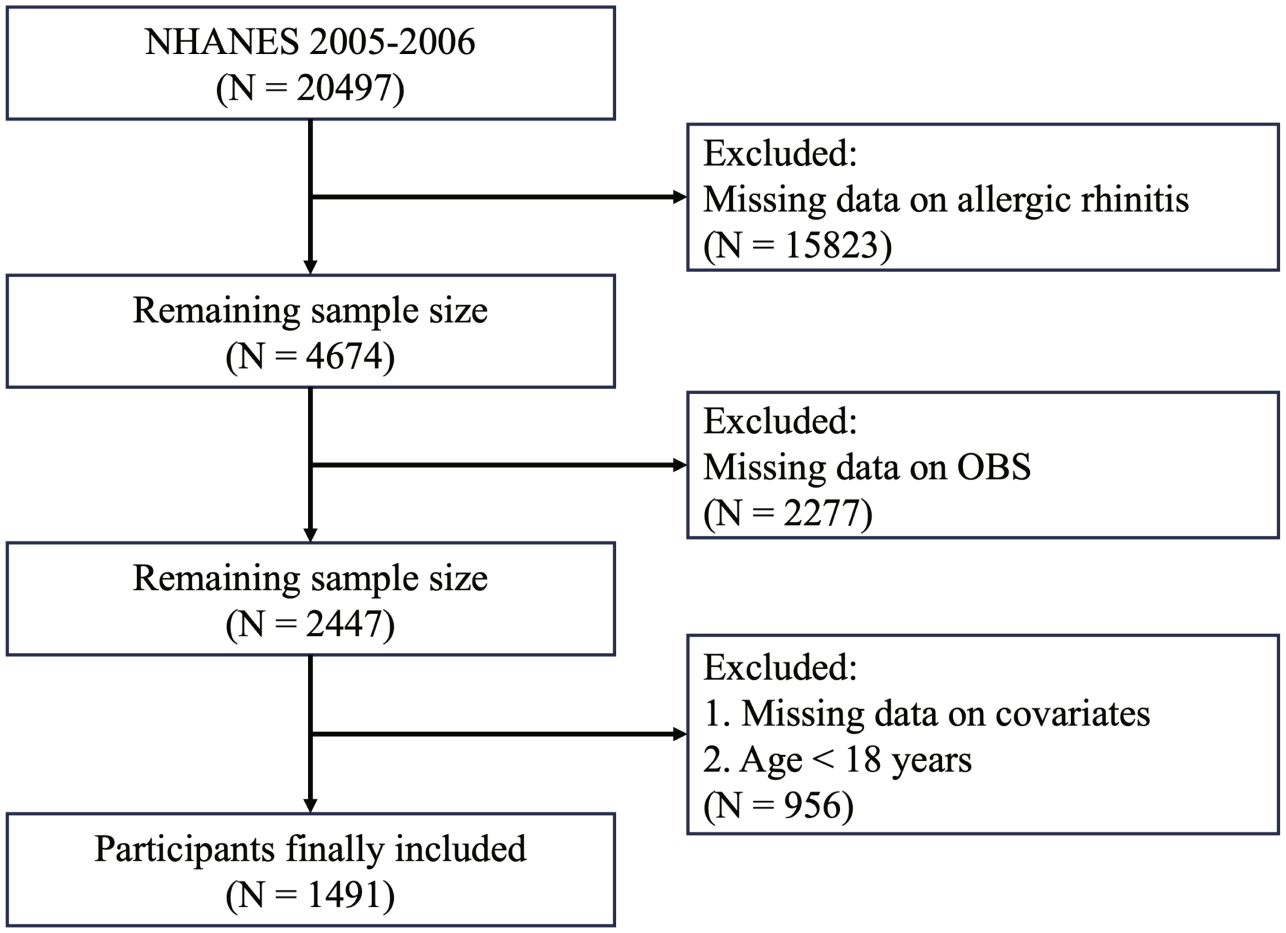
Participant selection flowchart.

Data were derived from the NHANES, a large-scale, cross-sectional study conducted by the U.S. National Institutes of Health (NIH) and the Centers for Disease Control and Prevention (CDC), designed to assess the nutritional and health status of the U.S. population. NHANES protocols were approved by the National Center for Health Statistics (NCHS) Research Ethics Review Board, and informed consent was obtained from all participants before participation. This study utilized publicly available data from the NHANES 2005–2006 cycle. Initially, a total of 20,497 participants were included. Participants were excluded if they met any of the following criteria: (1) missing AR data; (2) incomplete data for OBS calculation; or (3) missing data on included covariates. A detailed description of the inclusion and exclusion process is provided in Figure 2.

### Exposure assessment

The OBS was calculated by summing scores from 20 components, including 16 dietary and 4 lifestyle factors (13, 16). The dietary components were dietary fiber, carotene, riboflavin, niacin, vitamin B6, total folate, vitamin B12, vitamin C, vitamin E, calcium, magnesium, zinc, copper, selenium, total fat, and iron, while the four lifestyle components included physical activity, alcohol consumption, body mass index (BMI), and serum cotinine. Dietary data were obtained from the first 24-h dietary recall interview conducted in person at the Mobile Examination Center (MEC), and lifestyle information was collected through standardized questionnaires and laboratory tests. Based on prior evidence, total fat, alcohol, BMI, and cotinine were considered pro-oxidants, whereas the remaining components were treated as antioxidants. Antioxidants were scored from 0 to 2 (higher values indicating more favorable exposure), while pro-oxidants were inversely scored (2 to 0) to reflect risk.

Except for BMI, all other dietary and lifestyle components were stratified by sex where appropriate, to account for differences in daily energy intake and activity patterns between men and women. BMI was the only variable for which we did not apply gender-specific cut-offs, as the WHO adult classification uses the same thresholds for both sexes. Physical activity (antioxidant) was also divided into sex-specific tertiles and scored 0/1/2 from the lowest to the highest tertile within sex. Alcohol intake (pro-oxidant) was scored according to the Dietary Guidelines for Americans, 2020–2025 (9th ed.): non-drinkers = 2 points; moderate drinking = 1 point (≤14 g/day for women; ≤28 g/day for men); heavy drinking = 0 points (>14 g/day for women; >28 g/day for men). BMI was scored using WHO adult categories with the same thresholds for both sexes: <25.0 kg/m^2^ = 2 points; 25.0–29.9 kg/m^2^ = 1 point; ≥30.0 kg/m^2^ = 0 points. Serum cotinine (pro-oxidant) was divided into sex-specific tertiles and inversely scored (2/1/0 from the lowest to the highest tertile within sex). The total OBS was derived by summing all component scores, with higher scores representing more favorable oxidative balance. Full scoring details are provided in Supplementary Table S1.

### Ascertainment of outcomes

The diagnosis of AR was based on a combination of questionnaire data and serum allergen-specific IgE measurements. Relevant data were obtained from the 2005–2006 cycle of the NHANES, which included both allergy-related questionnaire responses and laboratory-based IgE test results for 19 specific allergens.

Two questionnaire items were used: AGQ030 (“During the past 12 months, have you had an episode of hay fever?”) and AGQ100 (“During the past 12 months, have you had a problem with sneezing, or a runny, or blocked nose when (2) did not have a cold or the flu?”). Serum-specific IgE levels were considered positive if they were ≥0.35 kU/L.

Participants were classified as having AR if they answered “yes” to either AGQ030 or AGQ100 and had a positive serum-specific IgE result. The control group included participants who answered “no” to both questions and had negative IgE results. Individuals who did not meet either criterion were excluded from the analysis (17, 18).

### Ascertainment of covariates

Covariates included in the analysis were age, gender, BMI, race/ethnicity, education, poverty-income ratio (PIR), smoking status, serum total IgE antibody, serum C-reactive protein (CRP), hypertension, diabetes, and cardiovascular disease (CVD). Race/ethnicity was categorized into five groups: Mexican American, Non-Hispanic Black, Non-Hispanic White, Other Hispanic, and Other Race. Education level was divided into three categories: above high school (college graduate or higher), high school or equivalent (high school diploma or GED), and below high school (including less than 9th grade, 9–11th grade, or no diploma). Smoking status was classified into three categories: never smokers (smoked fewer than 100 cigarettes in lifetime and currently do not smoke), former smokers (smoked at least 100 cigarettes in lifetime but no longer smoke), and current smokers (smoked at least 100 cigarettes in lifetime and currently smoke every day or on some days). Dietary supplement use was obtained from self-reported questionnaires, while total energy was assessed at the MEC using 24-h dietary recall interviews. Hypertension was defined by self-reported physician diagnosis, elevated average blood pressure (systolic ≥130 mmHg and/or diastolic ≥85 mmHg), or current use of antihypertensive medication. Diabetes was defined based on any of the following: fasting plasma glucose ≥7.0 mmol/L (126 mg/dL), 2-h oral glucose tolerance test (OGTT) result ≥11.1 mmol/L (200 mg/dL), hemoglobin A1c (HbA1c) ≥6.5%, or current use of insulin or oral hypoglycemic medications. CVD was defined using responses to the medical condition’s questionnaire. Participants were classified as having CVD if they reported ever being diagnosed by a physician with any of the following: coronary heart disease, congestive heart failure, stroke, myocardial infarction, or angina.

### Statistical analyses

Continuous variables were summarized as means with standard deviations (SD), and categorical variables as frequencies and percentages. The OBS was examined in three ways: per unit increase, per 1-SD increase, and as a quartile-based categorical variable, and all forms were applied in the models to evaluate its association with AR.

To assess the robustness and national representativeness of our finding, additional weighted analyses were performed in line with NHANES analytic guidelines. These accounted for the complex survey design by incorporating the 2-year MEC examination weight (WTMEC2YR), strata (SDMVSTRA), and primary sampling units (SDMVPSU). Baseline differences between groups after applying survey weights were assessed using standardized mean differences (SMDs).

Three multivariable logistic regression models were constructed. Model 1 was unadjusted. Model 2 was adjusted for age, sex, and race/ethnicity. Model 3 included additional adjustments for education level, PIR, serum CRP, smoking status, dietary supplement, total energy, hypertension, diabetes, and CVD. Multicollinearity was assessed using the variance inflation factor (VIF). Dose–response relationships were explored using restricted cubic spline (RCS) models based on Model 3, with knot placement optimized via Akaike information criterion (AIC). If nonlinearity was detected, associations on either side of the inflection point were estimated separately.

Subgroup analyses and interaction tests were conducted to explore effect modification across different population strata. To address the issue of multiple comparisons, the Benjamini–Hochberg method was applied to control the false discovery rate (FDR). To evaluate the robustness of the results, four sensitivity analyses were conducted. First, participants with asthma were excluded. Second, considering the potential associations between dietary supplement use and participants’ dietary habits or behaviors, participants who reported dietary supplement use were excluded. Third, dietary components of the OBS were energy-adjusted using the residual method based on total energy intake estimated from the first 24-h dietary recall interview conducted in person at the MEC. Fourth, alternative definitions of AR were applied, including defining AR as the presence of any sIgE ≥0.35 kU/L, and using a higher cutoff of ≥0.7 kU/L for sIgE.

All statistical tests were two-sided, with *p*-values <0.05 considered statistically significant. All analyses were performed using R software (version 4.5.0).

## Results

### Baseline characteristics

Among the 1,491 participants, 513 were classified as having AR, and 978 as controls. Table 1 presents the baseline characteristics of the study population stratified by AR status. Compared with the control group, participants with AR were significantly younger. There were no significant differences in gender distribution, BMI, serum CRP levels, or the prevalence of diabetes, hypertension, and CVD between the two groups. However, race/ethnicity, education level, PIR, and smoking status differed significantly between groups. Serum total IgE levels were markedly higher in the AR group. Participants with AR also exhibited significantly higher total and dietary OBS, whereas lifestyle OBS did not differ between groups. In addition, the AR group reported higher total energy intake and a greater prevalence of dietary supplement use. After weighting, the differences between groups in gender, BMI, PIR, serum CRP, hypertension, and diabetes were minimal (all SMD <0.1). Detailed results are presented in Supplementary Table S2.

**Table 1:** Baseline characteristics of study participants stratified by AR status.

Variables	Overall	Control	AR	*p*-value
*N*	1,491	978	513	
Age, years	48.22 (17.51)	49.81 (17.74)	45.19 (16.67)	<0.001
Gender (%)				0.493
Female	726 (48.7)	483 (49.4)	243 (47.4)	
Male	765 (51.3)	495 (50.6)	270 (52.6)	
BMI, kg/m^2^	28.79 (6.50)	28.75 (6.39)	28.84 (6.73)	0.802
Race/ethnicity (%)				0.020
Mexican American	258 (17.3)	190 (19.4)	68 (13.3)	
Non-Hispanic Black	291 (19.5)	181 (18.5)	110 (21.4)	
Non-Hispanic White	847 (56.8)	552 (56.4)	295 (57.5)	
Other Hispanic	45 (3.0)	27 (2.8)	18 (3.5)	
Other Race	50 (3.4)	28 (2.9)	22 (4.3)	
Education (%)				<0.001
Above high school	843 (56.5)	505 (51.6)	338 (65.9)	
Below high school	300 (20.1)	228 (23.3)	72 (14.0)	
High school or equivalent	348 (23.3)	245 (25.1)	103 (20.1)	
PIR	2.97 (1.60)	2.91 (1.57)	3.09 (1.66)	0.036
Serum total IgE, kU/L	122.83 (371.76)	42.70 (73.81)	275.43 (596.62)	<0.001
Serum CRP, mg/L	3.9 (7.5)	3.9 (6.3)	4.0 (9.3)	0.91
Total energy, kcal	2238.70 (1021.44)	2160.36 (962.93)	2388.06 (1110.34)	<0.001
Smoking status (%)				0.012
Former	396 (26.6)	277 (28.3)	119 (23.2)	
Now	302 (20.3)	208 (21.3)	94 (18.3)	
Never	793 (53.2)	493 (50.4)	300 (58.5)	
Hypertension (%)				0.114
Yes	592 (39.7)	403 (41.2)	189 (36.8)	
No	899 (60.3)	575 (58.8)	324 (63.2)	
Diabetes (%)				0.395
Yes	191 (12.8)	131 (13.4)	60 (11.7)	
No	1,300 (87.2)	847 (86.6)	453 (88.3)	
CVD (%)				0.281
Yes	128 (8.6)	90 (9.2)	38 (7.4)	
No	1,363 (91.4)	888 (90.8)	475 (92.6)	
Dietary supplement (%)				0.041
Yes	755 (50.6)	476 (48.7)	279 (54.4)	
No	736 (49.4)	502 (51.3)	234 (45.6)	
Total OBS	20.74 (6.87)	20.25 (7.06)	21.67 (6.41)	<0.001
Dietary OBS	17.07 (6.48)	16.60 (6.63)	17.96 (6.08)	<0.001
Lifestyle OBS	3.67 (1.49)	3.65 (1.49)	3.71 (1.50)	0.497

AR, allergic rhinitis; PIR, poverty-income ratio; CRP, serum C-reactive protein; CVD, cardiovascular disease; OBS, oxidative balance score.

### Association between OBS and allergic rhinitis

VIF analysis revealed no evidence of multicollinearity, with all VIF values below 5 (Supplementary Table S3). Table 2 shows the associations between OBS and AR across three logistic regression models.

**Table 2:** Association between total, dietary, and lifestyle OBS and AR.

Characteristic	Model 1		Model 2		Model 3	
	OR (95% CI)	*p*	OR (95% CI)	*p*	OR (95% CI)	*p*
Total OBS	1.03 (1.01, 1.04)	<0.001	1.03 (1.01, 1.05)	<0.001	1.02 (1.01, 1.04)	0.003
Total OBS (per SD)	1.21 (1.09, 1.35)	<0.001	1.24 (1.11, 1.39)	<0.001	1.19 (1.06, 1.34)	0.003
Total OBS quartile						
Q1	Ref.	—	Ref.	—	Ref.	—
Q2	1.46 (1.07, 2.00)	0.018	1.5 (1.09, 2.07)	0.012	1.41 (1.02, 1.95)	0.037
Q3	1.58 (1.16, 2.17)	0.004	1.64 (1.19, 2.26)	0.003	1.52 (1.10, 2.11)	0.012
Q4	1.76 (1.30, 2.41)	<0.001	1.87 (1.36, 2.58)	<0.001	1.67 (1.20, 2.33)	0.003
*p* for trend		<0.001		<0.001		0.003
Dietary OBS	1.03 (1.01, 1.05)	<0.001	1.03 (1.02, 1.05)	<0.001	1.03 (1.01, 1.04)	0.003
Dietary OBS (per SD)	1.22 (1.10, 1.36)	<0.001	1.24 (1.11, 1.39)	<0.001	1.19 (1.06, 1.34)	0.003
Dietary OBS quartile						
Q1	Ref.	—	Ref.	—	Ref.	—
Q2	1.44 (1.06, 1.97)	0.022	1.43 (1.04, 1.96)	0.028	1.37 (0.99, 1.89)	0.055
Q3	1.56 (1.15, 2.14)	0.005	1.56 (1.13, 2.15)	0.006	1.47 (1.06, 2.03)	0.020
Q4	1.72 (1.26, 2.35)	<0.001	1.75 (1.28, 2.41)	<0.001	1.60 (1.16, 2.22)	0.005
*p* for trend		<0.001		<0.001		0.005
Lifestyle OBS	1.00 (0.93, 1.06)	0.900	1.04 (0.97, 1.11)	0.300	1.01 (0.93, 1.08)	0.900
Lifestyle OBS (per SD)	0.99 (0.89, 1.10)	0.900	1.06 (0.95, 1.18)	0.300	1.01 (0.90, 1.14)	0.900
Lifestyle OBS quartile						
Q1	Ref.	—	Ref.	—	Ref.	—
Q2	0.94 (0.70, 1.27)	0.700	0.98 (0.72, 1.33)	0.900	0.89 (0.65, 1.22)	0.500
Q3	0.94 (0.70, 1.27)	0.700	1.09 (0.80, 1.48)	0.600	0.98 (0.71, 1.35)	0.900
Q4	0.95 (0.70, 1.28)	0.700	1.09 (0.80, 1.49)	0.600	0.96 (0.69, 1.33)	0.800
*p* for trend		0.734		0.466		0.945

Model 1: No covariates were adjusted.

Model 2: Adjusted for age, gender, race/ethnicity.

Model 3: Adjusted for age, gender, race/ethnicity, education level, PIR, serum CRP, smoking status, hypertension, diabetes, CVD, and dietary supplement use.

**Figure 3:**
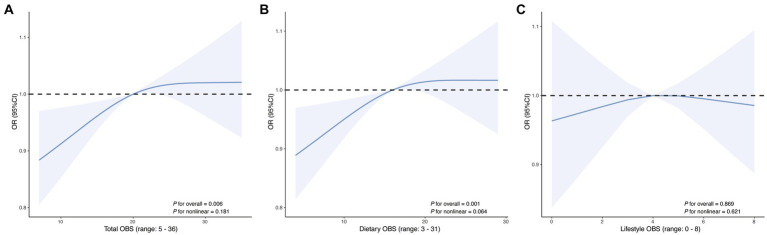
Restricted cubic spline curves showing the association of **(A)** total OBS, **(B)** dietary OBS, and **(C)** lifestyle OBS with AR. The solid line represents the estimated OR, and the shaded area indicates the 95% CI. The *x*-axis displays the observed range of each OBS score.

For total OBS, significant positive associations with AR were consistently observed. In the fully adjusted Model 3, each unit increase in total OBS was associated with a 2% higher likelihood of AR (OR = 1.02; 95% CI: 1.01, 1.04; *p* = 0.003). When expressed per SD increase, total OBS was associated with a 19% higher likelihood of AR (OR = 1.19; 95% CI: 1.06, 1.34; *p* = 0.003). Quartile-based analysis indicated a dose–response relationship, with participants in the highest OBS quartile (Q4) having significantly increased odds of AR compared to the lowest quartile (Q1) (OR = 1.67; 95% CI: 1.20, 2.33; *p* = 0.003). The trend across quartiles was statistically significant (*p* for trend = 0.003). Dietary OBS demonstrated a similar pattern. In Model 3, each unit increase in dietary OBS corresponded to a 3% higher likelihood of AR (OR = 1.03; 95% CI: 1.01, 1.04; *p* = 0.003). When expressed per SD increase, dietary OBS was associated with a 19% higher likelihood of AR (OR = 1.19; 95% CI: 1.06, 1.34; *p* = 0.003). Quartile analysis reinforced this association, with the highest dietary OBS quartile showing increased odds (OR = 1.60; 95% CI: 1.16, 2.22; *p* = 0.005) and a significant trend (*p* for trend = 0.005). Lifestyle OBS did not show significant associations with AR in either unit (OR = 1.01; 95% CI: 0.93–1.08; *p* = 0.900), per SD (OR = 1.01; 95% CI: 0.90–1.14; *p* = 0.900), or quartile analyses (all *p* > 0.05). RCS analyses (Figure 3) revealed significant linear relationships between OBS and AR, including total OBS (*p* for overall = 0.006) and dietary OBS (*p* for overall = 0.001).

For total OBS, significant positive associations with AR were also observed after applying survey weights. In the fully adjusted Model 3, each unit increase in total OBS was associated with a 2% higher likelihood of AR (OR = 1.02; 95% CI: 1.00, 1.03; *p* = 0.047). When expressed per SD increase, total OBS was associated with a 12% higher likelihood of AR (OR = 1.12; 95% CI: 1.00, 1.26; *p* = 0.047). Quartile-based analysis further supported a dose–response relationship, with participants in the highest OBS quartile (Q4) having significantly increased odds of AR compared to the lowest quartile (Q1) (OR = 1.65; 95% CI: 1.08, 2.51; *p* = 0.029). The trend across quartiles remained statistically significant (*p* for trend = 0.017). For dietary OBS and lifestyle OBS, no significant associations with AR were observed after applying survey weights. Detailed results are presented in Supplementary Table S4.

### Sensitivity analysis

In sensitivity analyses, the results were largely consistent with the primary findings. First, after excluding participants with asthma, total OBS and dietary OBS were still robustly associated with AR, with clear dose–response relationships across quartiles (*p* for trend <0.001; Supplementary Table S5). Second, after excluding participants who reported dietary supplement use yielded comparable results, reinforcing the robustness of the associations (Supplementary Table S6). Third, dietary components of the OBS were energy-adjusted using the residual method (assignment scheme shown in Supplementary Table S7). After energy adjustment, both total and dietary OBS remained significantly associated with AR with consistent dose–response patterns, whereas lifestyle OBS showed no significant association (Supplementary Table S8). Finally, applying alternative definitions of AR (≥0.35 kU/L or ≥0.7 kU/L for sIgE positivity) did not materially change the results, and positive associations between OBS and AR were consistently observed (Supplementary Table S9).

### Subgroup analyses

**Figure 4:**
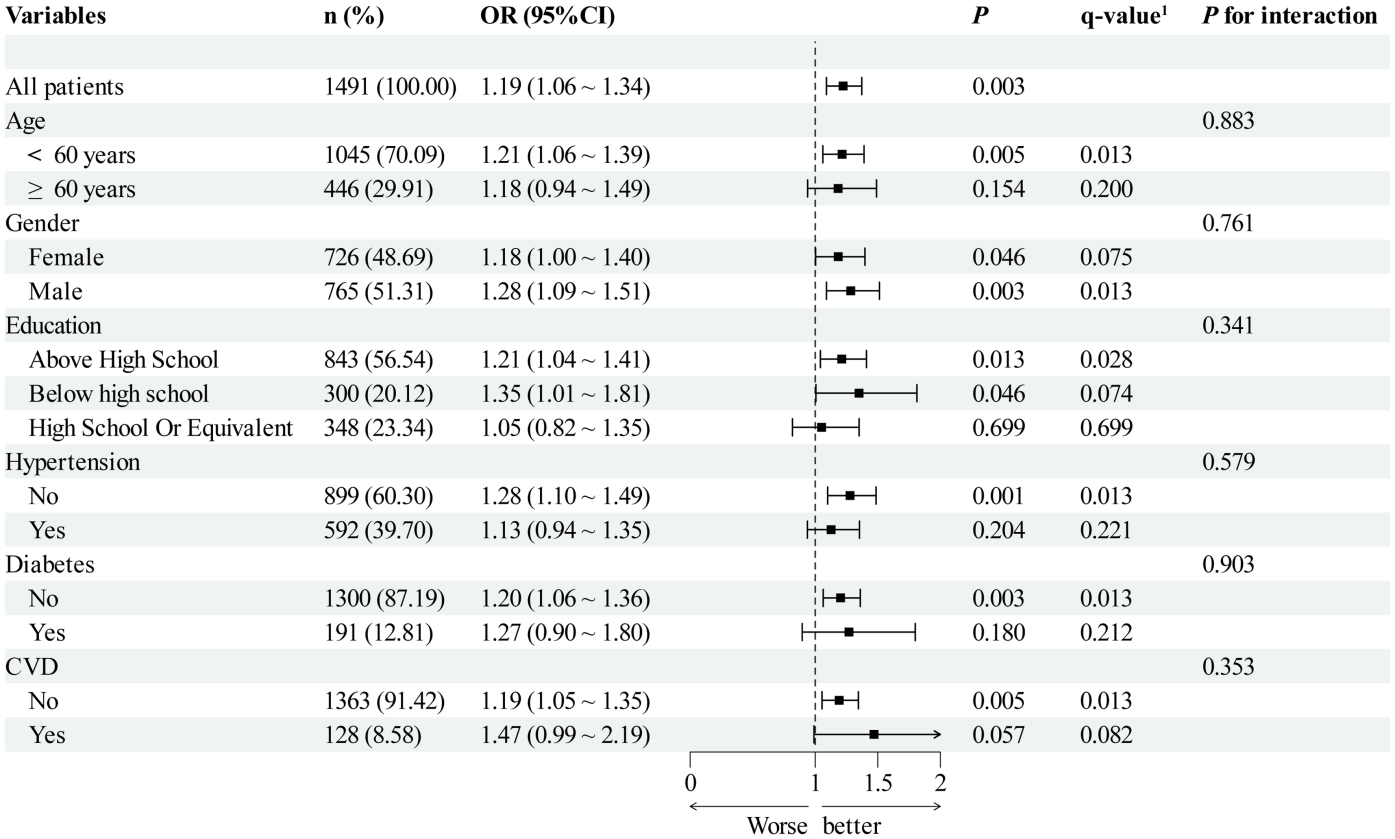
Subgroup analyses of the association between the total OBS (per SD) and AR. *q*-value: *p*-values for subgroups were adjusted for multiple comparisons using the Benjamini–Hochberg method to control the false discovery rate.

Exploratory subgroup analyses were performed to assess whether the association between total OBS (per SD) and AR was consistent across demographic and clinical subgroups (Figure 4). After controlling the FDR, the positive association was largely consistent across subgroups. The association appeared stronger in younger participants, males, and those with lower educational attainment. However, none of the interaction terms reached statistical significance (all *p* for interaction >0.05).

## Discussion

This study, using nationally representative data from NHANES 2005–2006, demonstrated a positive association between the OBS and AR. In unweighted analyses, higher total and dietary OBS were associated with greater odds of AR when modeled as continuous and quartile-based exposures, and these associations persisted after multivariable adjustment for sociodemographic, behavioral, and clinical covariates. A monotonic dose–response pattern across quartiles was evident, and restricted cubic spline analyses supported linear relationships without evidence of nonlinearity. In survey-weighted models, the association remained for total OBS, whereas dietary and lifestyle OBS were not statistically significant. Findings were broadly consistent across prespecified sensitivity analyses (including exclusion of participants with asthma or dietary supplement use, energy adjustment of dietary components, and alternative AR definitions). In exploratory subgroup analyses, after controlling the FDR, the positive association was largely consistent across subgroups.

To our knowledge, only one prior study has examined the association between a composite oxidative balance index and AR in a general population. Hwang et al. (19) reported a positive association between oxidative balance and AR using nationally representative Korean data, providing initial epidemiological evidence linking redox status to allergic conditions. However, their research was presented as a brief research letter with limited methodological elaboration. Unlike their study, our analysis offers a more comprehensive and rigorous evaluation. We incorporated a broader set of covariates and further corroborated our findings through sensitivity strategies, including energy adjustment of dietary components via the residual method, exclusion of participants with asthma, and application of alternative AR definitions. As part of this process, we also evaluated a symptom-only definition; after applying exclusion criteria, the retained sample yielded estimates consistent with the primary analysis. More broadly, the direction of association was preserved across alternative definitions (symptom-only, sIgE-only, and varying sIgE thresholds), although definition-specific differences in sensitivity and specificity should be considered when extrapolating these results to other populations. These methodological enhancements strengthen the robustness and generalizability of our findings and provide deeper insights into the complex relationship between oxidative balance and AR.

Although oxidative stress has long been implicated in the pathophysiology of allergic diseases, prior studies have predominantly relied on individual biomarkers such as malondialdehyde or antioxidant enzymes (e.g., superoxide dismutase, glutathione peroxidase) to infer redox status (11, 20). Observational studies have reported that higher intake of dietary antioxidants such as vitamins C and E, carotenoids, and flavonoids may be inversely associated with allergic symptoms (21), while pro-oxidant exposures such as smoking and obesity are known to exacerbate airway inflammation (22, 23). While existing literature has highlighted the role of oxidative stress in allergic diseases and proposed several mechanistic pathways, most prior evidence has relied on either biochemical markers of oxidative damage or focused on individual dietary or environmental exposures. Although these studies provide important insights, they often assess oxidant or antioxidant factors in isolation, without quantitatively integrating the overall oxidative-antioxidant profile at the population level. The OBS, originally developed by Goodman et al. (24), addresses this gap by combining multiple dietary and lifestyle factors into a single, comprehensive index. Previous studies have demonstrated that higher OBS is associated with a lower risk of CVD, certain cancers, and metabolic disorders (25–28). Our findings extend the application of this composite score to allergic conditions, supporting the relevance of redox balance in immune-mediated diseases such as AR. Moreover, in sensitivity analyses we energy-adjusted the dietary components using the residual method and recalculated an energy-adjusted OBS; re-estimation under this scheme yielded concordant effect estimates and did not materially alter the inference, thereby strengthening the robustness of our main conclusion.

Although a higher OBS generally reflects a favorable oxidative-antioxidative profile, our counterintuitive finding of a positive association with AR may, at least in part, reflect behavioral and measurement factors. We speculate that reverse causation plays a role—individuals with AR may modify diet or begin supplement use after symptom onset or diagnosis, thereby increasing dietary OBS (29). Some antioxidant-rich dietary patterns may also co-occur with allergenic food exposures or other unmeasured confounders. Moreover, diet and supplement intake can change rapidly and are captured by a single 24-h recall, whereas lifestyle components—tobacco-smoke exposure (cotinine), BMI, physical activity, and alcohol—typically evolve more slowly and, across studies, show heterogeneous and often modest associations with AR (30–32); this slower timescale and heterogeneity may attenuate the composite lifestyle signal relative to diet-driven variation. Finally, reliance on a single 24 h recall introduces within-person variability and measurement error in estimating usual intake; energy adjustment can mitigate but not eliminate such error. Taken together, these considerations provide a plausible explanation for why dietary OBS showed a stronger association than lifestyle OBS and help contextualize the overall positive association observed here. Additionally, Table 1 shows higher total and dietary OBS in the AR group; this descriptive difference, directionally consistent with the model-based results, may likewise reflect reverse causation, short-term dietary variability captured by a single 24-h recall, and residual confounding.

This study has several strengths, which also represent our strategies to minimize potential bias. First, by utilizing data from the NHANES 2005–2006 cycle—a nationally representative survey of the U.S. population—and incorporating survey weights, we ensured external validity and generalizability. Second, by adjusting for a wide range of sociodemographic, behavioral, and clinical covariates, including multiple chronic diseases, we improved internal validity and helped isolate the independent association between oxidative balance and allergic rhinitis. Third, to ensure robustness, we conducted four sensitivity analyses (energy adjustment, exclusion of participants with asthma, exclusion of supplement users, and alternative AR definitions), and the consistent findings across these analyses support the stability and reliability of our results. Fourth, in exploratory subgroup analyses, we applied FDR correction for multiple testing, further enhancing robustness. Collectively, these strategies both reduce the risk of bias and highlight the methodological strengths of our study.

However, several limitations warrant attention. First, the cross-sectional design precludes causal inference, and reverse causality cannot be ruled out; individuals with AR may have modified their diet or supplement use, potentially influencing OBS. Second, the OBS does not capture all sources of oxidative stress, such as environmental pollution, aeroallergens, occupational exposures, or genetic variation in antioxidant enzymes, and NHANES lacks information on seasonality, geographic variation, and other environmental covariates that may influence both diet and AR. Third, dietary variables were derived from a single 24-h recall, which may not accurately reflect habitual intake and could introduce measurement error in OBS calculation. Fourth, although we adjusted for dietary supplement use in sensitivity analyses, residual confounding related to supplement intake and other unmeasured behaviors cannot be excluded. Lastly, while we reported associations per unit, SD, and quartiles of OBS to improve interpretability, residual confounding may still exist despite adjustment for extensive covariates.

## Conclusion

In a nationally representative sample, higher oxidative balance score (OBS) was associated with greater odds of allergic rhinitis after extensive adjustment, with a monotonic dose–response; signals were stronger for dietary than lifestyle components, and persisted for total OBS in survey-weighted models. Importantly, this study provides one of the first population-based evaluations of OBS in relation to allergic rhinitis, demonstrating the utility of OBS as a composite marker in nutritional immunology. By integrating dietary and lifestyle exposures with rigorous sensitivity analyses, our findings contribute novel evidence to the field and highlight the relevance of redox balance in allergic disease epidemiology. These findings suggest diet-related redox balance is relevant to AR at the population level but may be shaped by behavior change and measurement error; they therefore do not support escalating antioxidant supplementation. Future work should establish causality using longitudinal designs that incorporate season/region and environmental exposures, integrate objective redox biomarkers, and, where feasible, test food-based interventions.

## Data Availability

The datasets generated and analyzed in the current study are available at NHANES website: https://www.cdc.govnchsnhanesindex.html.
