# Supplementary material for: Association between oxidative balance score and allergic rhinitis in American adults: a cross-sectional study
Source: Front Nutr. 2025 Sep 25;12:1655316. doi: 10.3389/fnut.2025.1655316 (PMC12509687; doi:10.3389/fnut.2025.1655316)
Supplement: Supplementary file 1 [file Table_1.DOCX]

**Supplementary material**

**Table S1. OBS Scoring Scheme**

**Table S2. Weighted Baseline Characteristics of Participants Stratified by Allergic Rhinitis Status**

**Table S3. Variance Inflation Factor (VIF) Analysis for Multicollinearity Diagnosis**

**Table S4. Survey-Weighted Associations Between Total, Dietary, and Lifestyle OBS and Allergic Rhinitis**

**Table S5. Sensitivity Analysis 1: Associations Between Total and Dietary OBS and Allergic Rhinitis After Excluding Participants With Asthma**

**Table S6. Sensitivity Analysis 2: Associations Between Total and Dietary OBS and Allergic Rhinitis After Excluding Participants Reporting Dietary Supplement Use**

**Table S7. OBS Scoring Scheme After Energy Adjustment Using the Residual Method**

**Table S8. Sensitivity Analysis 3: Associations Between Total, Dietary, and Lifestyle OBS and Allergic Rhinitis After Energy Adjustment**

**Table S9. Sensitivity Analysis 4: Associations Between Total and Dietary OBS and Allergic Rhinitis Under Alternative Definitions of Allergic Rhinitis**

**Table S1. OBS Scoring Scheme**

| **OBS components** | **Property** | **Male** | | | **Female** | | |
| --- | --- | --- | --- | --- | --- | --- | --- |
|  |  | 0 | 1 | 2 | 0 | 1 | 2 |
| Dietary fiber (g/d) | A | <13.67 | 13.67-20.65 | ≥20.65 | <11.25 | 11.25-16.22 | ≥16.22 |
| Carotene (RE/d) | A | <56.84 | 56.84-182.31 | ≥182.31 | <57.82 | 57.82-206.92 | ≥206.92 |
| Riboflavin (mg/d) | A | <2.01 | 2.01-2.84 | ≥2.84 | <1.47 | 1.47-2.12 | ≥2.12 |
| Niacin (mg/d) | A | <23.72 | 23.72-33.29 | ≥33.29 | <16.47 | 16.47-23.68 | ≥23.68 |
| Vitamin B6 (mg/d) | A | <1.81 | 1.81-2.64 | ≥2.64 | <1.31 | 1.31-1.92 | ≥1.92 |
| Total folate (mcg/d) | A | <355.50 | 355.50-521.00 | ≥521.00 | <265.83 | 265.83-386.00 | ≥386.00 |
| Vitamin B12 (mcg/d) | A | <4.26 | 4.26-6.96 | ≥6.96 | <2.79 | 2.79-5.09 | ≥5.09 |
| Vitamin C (mg/d) | A | <48.73 | 48.73-112.82 | ≥112.82 | <43.93 | 43.93-97.70 | ≥97.70 |
| Vitamin E (ATE) (mg/d) | A | <5.85 | 5.85-8.86 | ≥8.86 | <4.62 | 4.62-7.22 | ≥7.22 |
| Calcium (mg/d) | A | <748.33 | 748.33-1149.67 | ≥1149.67 | <595.83 | 595.83-925.00 | ≥925.00 |
| Magnesium (mg/d) | A | <272.50 | 272.50-367.50 | ≥367.50 | <211.50 | 211.50-291.67 | ≥291.67 |
| Zinc (mg/d) | A | <11.30 | 11.30-15.85 | ≥15.85 | <7.71 | 7.71-11.50 | ≥11.50 |
| Copper (mg/d) | A | <1.22 | 1.22-1.66 | ≥1.66 | <0.94 | 0.94-1.29 | ≥1.29 |
| Selenium (mcg/d) | A | <102.00 | 102.00-143.93 | ≥143.93 | <75.57 | 75.57-103.75 | ≥103.75 |
| Total fat (g/d) | P | ≥108.75 | 72.28-108.75 | <72.28 | ≥76.12 | 52.23-76.12 | <52.23 |
| Iron (mg/d) | P | ≥20.76 | 14.66-20.76 | <14.66 | ≥15.46 | 10.65-15.46 | <10.65 |
| Physical activity(MET-minute/week) | A | ＜252 | 252-822.11 | ≥822.11 | ＜252 | 252-756 | ≥756 |
| Alcohol (g/d) | P | ＞28 | 0-28 | 0 | ＞14 | 0-14 | 0 |
| BMI (kg/m^2^) | P | ≥30.0 | 25.0-29.9 | ＜25.0 | ≥30.0 | 25.0-29.9 | ＜25.0 |
| Cotinine (ng/mL) | P | ≥1.25 | 0.03-1.25 | ＜0.03 | ≥0.11 | 0.02-0.11 | ＜0.02 |

OBS: oxidative balance score; A: antioxidant; P: prooxidant; RE: retinol equivalent; ATE: alpha-tocopherol equivalent; MET: metabolic equivalent; BMI: body mass index

**Table S2. Weighted Baseline Characteristics of Participants Stratified by Allergic Rhinitis Status**

|  | **Overall** | **Control** | **Allergic rhinitis** | **SMD** |
| --- | --- | --- | --- | --- |
| **N** | 77569896.53 | 49429945.17 | 28139951.36 |  |
| **Age, years** | 46.09 (15.78) | 47.66 (16.19) | 43.35 (14.63) | 0.279 |
| **Gender (%)** |  |  |  | 0.058 |
| *female* | 38694075.7 (49.9) | 25173700.6 (50.9) | 13520375.0 (48.0) |  |
| *male* | 38875820.9 (50.1) | 24256244.5 (49.1) | 14619576.3 (52.0) |  |
| ***BMI, kg/m^2^*** | 28.68 (6.59) | 28.74 (6.55) | 28.57 (6.66) | 0.026 |
| **Race/ethnicity (%)** |  |  |  | 0.130 |
| *Mexican American* | 4784777.7 (6.2) | 3468601.9 (7.0) | 1316175.8 (4.7) |  |
| *Non-Hispanic Black* | 6652577.8 (8.6) | 4167456.0 (8.4) | 2485121.8 (8.8) |  |
| *Non-Hispanic White* | 60221617.3 (77.6) | 38421014.0 (77.7) | 21800603.3 (77.5) |  |
| *Other Hispanic* | 2544531.3 (3.3) | 1538268.0 (3.1) | 1006263.3 (3.6) |  |
| *Other Race* | 3366392.4 (4.3) | 1834605.2 (3.7) | 1531787.2 (5.4) |  |
| **Education (%)** |  |  |  | 0.302 |
| *above high school* | 50320239.9 (64.9) | 29622227.8 (59.9) | 20698012.1 (73.6) |  |
| *Below high school* | 9651463.9 (12.4) | 7359191.1 (14.9) | 2292272.8 (8.1) |  |
| *high school or equivalent* | 17598192.7 (22.7) | 12448526.3 (25.2) | 5149666.4 (18.3) |  |
| **PIR** | 3.36 (1.53) | 3.33 (1.51) | 3.43 (1.58) | 0.069 |
| **Serum total IgE, kU/L** | 102.83 (296.71) | 34.84 (52.69) | 222.19 (464.33) | 0.567 |
| **Serum CRP, mg/L** | 3.8 (8.1) | 3.8 (6.0) | 3.8 (10.8) | 0.009 |
| **Total energy, kcal** | 2309.44 (1047.89) | 2245.45 (1008.05) | 2421.85 (1106.47) | 0.167 |
| **Smoking status (%)** |  |  |  | 0.215 |
| *former* | 20678872.0 (26.7) | 14202744.2 (28.7) | 6476127.9 (23.0) |  |
| *now* | 41369256.4 (53.3) | 24458507.5 (49.5) | 16910748.8 (60.1) |  |
| *never* | 15521768.2 (20.0) | 10768693.5 (21.8) | 4753074.7 (16.9) |  |
| **Hypertension (%)** |  |  |  | 0.086 |
| *Yes* | 27873995.0 (35.9) | 18497338.7 (37.4) | 9376656.3 (33.3) |  |
| *No* | 49695901.5 (64.1) | 30932606.5 (62.6) | 18763295.1 (66.7) |  |
| **Diabetes (%)** |  |  |  | 0.043 |
| *Yes* | 7639229.7 (9.8) | 5095718.9 (10.3) | 2543510.8 (9.0) |  |
| *No* | 69930666.9 (90.2) | 44334226.3 (89.7) | 25596440.6 (91.0) |  |
| **CVD (%)** |  |  |  | 0.101 |
| *Yes* | 5160532.4 (6.7) | 3728729.1 (7.5) | 1431803.3 (5.1) |  |
| *No* | 72409364.1 (93.3) | 45701216.0 (92.5) | 26708148.0 (94.9) |  |
| **Dietary supplement(%)** |  |  |  | 0.028 |
| *Yes* | 42078877.2 (54.2) | 26566928.6 (53.7) | 15511948.6 (55.1) |  |
| *No* | 35491019.4 (45.8) | 22863016.6 (46.3) | 12628002.8 (44.9) |  |
| **Total OBS** | 21.21 (7.10) | 20.43 (7.19) | 21.89 (6.89) | 0.021 |
| **Dietary OBS** | 16.91 (6.75) | 16.44 (6.82) | 17.44 (6.58) | 0.049 |
| **Lifestyle OBS** | 4.41 (1.58) | 4.38 (1.58) | 4.45 (1.58) | 0.039 |

**Table S3. Variance Inflation Factor (VIF) Analysis for Multicollinearity Diagnosis**

| **Variable** | **VIF** |
| --- | --- |
| Age | 1.466137 |
| Gender male | 1.038576 |
| Race/ethnicity Non-Hispanic Black | 2.102508 |
| Race/ethnicity Non-Hispanic White | 2.486280 |
| Race/ethnicity Other Hispanic | 1.189255 |
| Race/ethnicity Other Race | 1.243558 |
| Education Below high school | 1.381753 |
| Education High school or equivalent | 1.156939 |
| PIR | 1.269640 |
| CRP | 1.026029 |
| Smoking never | 1.546734 |
| Smoking now | 1.577544 |
| Hypertension | 1.319480 |
| Diabetes | 1.141233 |
| CVD | 1.116590 |
| **Dietary supplement** | 1.148432 |

**Table S4. Survey-Weighted Associations Between Total, Dietary, and Lifestyle OBS and Allergic Rhinitis**

| **Characteristic** | **Model 1** | | **Model 2** | | **Model 3** | |
| --- | --- | --- | --- | --- | --- | --- |
|  | OR(95% CI) | *P* | OR(95% CI) | *P* | OR(95% CI) | *P* |
| **Total OBS** | 1.02 (1.01, 1.04) | 0.006 | 1.02 (1.01, 1.04) | 0.008 | 1.02 (1.00, 1.03) | 0.047 |
| **Total OBS (per SD)** | 1.16 (1.05, 1.29) | 0.006 | 1.17 (1.05, 1.30) | 0.008 | 1.12 (1.00, 1.26) | 0.047 |
| **Total OBS quartile** |  |  |  |  |  |  |
| Q1 | Ref. | - | Ref. | - | Ref. | - |
| Q2 | 1.34 (0.84, 2.12) | 0.200 | 1.34 (0.81, 2.21) | 0.200 | 1.34 (0.78, 2.30) | 0.200 |
| Q3 | 1.39 (1.00, 1.95) | 0.052 | 1.37 (0.98, 1.91) | 0.06 | 1.37 (0.95, 1.97) | 0.078 |
| Q4 | 1.62 (1.15, 2.28) | 0.009 | 1.64 (1.12, 2.41) | 0.018 | 1.65 (1.08, 2.51) | 0.029 |
| *P* for trend |  | 0.004 |  | 0.008 |  | 0.017 |
| **Dietary OBS** | 1.02 (1.00, 1.04) | 0.018 | 1.02 (1.01, 1.04) | 0.022 | 1.02 (0.99, 1.04) | 0.140 |
| **Dietary OBS (per SD)** | 1.16 (1.03, 1.31) | 0.018 | 1.16 (1.03, 1.31) | 0.022 | 1.12 (0.97, 1.28) | 0.140 |
| **Dietary OBS quartile** |  |  |  |  |  |  |
| Q1 | Ref. | - | Ref. | - | Ref. | - |
| Q2 | 1.29 (0.74, 2.25) | 0.300 | 1.24 (0.69, 2.22) | 0.400 | 1.24 (0.66, 2.34) | 0.400 |
| Q3 | 1.35 (0.99, 1.84) | 0.057 | 1.31 (0.96, 1.77) | 0.078 | 1.30 (0.92, 1.84) | 0.110 |
| Q4 | 1.54 (1.06, 2.25) | 0.027 | 1.52 (1.03, 2.26) | 0.039 | 1.52 (0.99, 2.36) | 0.056 |
| *P* for trend |  | 0.013 |  | 0.020 |  | 0.026 |
| **Lifestyle OBS** | 1.03 (0.93, 1.13) | 0.600 | 1.04 (0.94, 1.16) | 0.400 | 1.00 (0.87, 1.16) | ＞0.900 |
| **Lifestyle OBS (per SD)** | 1.04 (0.89, 1.21) | 0.600 | 1.07 (0.90, 1.26) | 0.400 | 1.01 (0.80, 1.26) | ＞0.900 |
| **Lifestyle OBS quartile** |  |  |  |  |  |  |
| Q1 | Ref. | - | Ref. | - | Ref. | - |
| Q2 | 0.94 (0.69, 1.27) | 0.700 | 1.00 (0.75, 1.34) | 0.900 | 0.88 (0.53, 1.49) | 0.400 |
| Q3 | 1.07 (0.63, 1.84) | 0.800 | 1.17 (0.65, 2.08) | 0.600 | 1.00 (0.29, 3.36) | 0.900 |
| Q4 | 1.03 (0.74, 1.43) | 0.800 | 1.11 (0.75, 0.99) | 0.600 | 0.89 (0.39, 2.05) | 0.600 |
| *P* for trend |  | 0.700 |  | 0.500 |  | 0.792 |

Model 1: no covariates were adjusted;

Model 2: Adjusted for age, gender, race/ethnicity;

Model 3: Adjusted for age, gender, race/ethnicity, education level, PIR, serum CRP, smoking status, hypertension, diabetes, CVD, and dietary supplement use.

**Table S5. Sensitivity Analysis 1: Associations Between Total and Dietary OBS and Allergic Rhinitis After Excluding Participants With Asthma**

| Characteristic | **Sensitivity analysis 1** | |
| --- | --- | --- |
|  | OR(95% CI) | *P* |
| Total OBS | 1.03(1.01, 1.05) | <0.001 |
| Total OBS (per SD) | 1.25(1.10, 1.43) | <0.001 |
| Total OBS quartile |  |  |
| Q1 | Ref. | - |
| Q2 | 1.57 (1.09, 2.26) | 0.017 |
| Q3 | 1.60 (1.10, 2.33) | 0.014 |
| Q4 | 1.99 (1.37, 2.90) | <0.001 |
| *P* for trend |  | <0.001 |
| Dietary OBS | 1.03(1.01, 1.05) | <0.001 |
| Dietary OBS (per SD) | 1.23 (1.09, 1.41) | <0.001 |
| Dietary OBS quartile |  |  |
| Q1 | Ref. | - |
| Q2 | 1.44 (1.00, 2.08) | 0.051 |
| Q3 | 1.53 (1.06, 2.22) | 0.025 |
| Q4 | 1.85 (1.29, 2.69) | 0.001 |
| *P* for trend |  | <0.001 |

Sensitivity analysis 1: Models were adjusted for age, gender, race/ethnicity, education level, PIR, serum CRP, smoking status, hypertension, diabetes, CVD, and dietary supplement use.

**Table S6. Sensitivity Analysis 2: Associations Between Total and Dietary OBS and Allergic Rhinitis After Excluding Participants Reporting Dietary Supplement Use**

| Characteristic | Sensitivity analysis 2 | |
| --- | --- | --- |
|  | OR(95% CI) | *P* |
| Total OBS | 1.02 (1.00, 1.04) | 0.047 |
| Total OBS (per SD) | 1.18 (1.00, 1.39) | 0.047 |
| Total OBS quartile |  |  |
| Q1 | Ref. | - |
| Q2 | 1.18 (0.73, 1.92) | 0.320 |
| Q3 | 1.47 (0.92, 2.37) | 0.110 |
| Q4 | 1.54 (1.06, 2.26) | 0.046 |
| *P* for trend |  | 0.043 |
| Dietary OBS | 1.02 (1.01, 1.04) | 0.005 |
| Dietary OBS (per SD) | 1.18 (1.05, 1.33) | 0.005 |
| Dietary OBS quartile |  |  |
| Q1 |  |  |
| Q2 | 1.36 (0.99, 1.88) | 0.062 |
| Q3 | 1.44 (1.04, 2.00) | 0.027 |
| Q4 | 1.56 (1.12, 2.16) | 0.008 |
| *P* for trend |  | 0.010 |

Sensitivity analysis 2: Models were adjusted for age, gender, race/ethnicity, education level, PIR, serum CRP, smoking status, hypertension, diabetes, and CVD.

**Table S7. OBS Scoring Scheme After Energy Adjustment Using the Residual Method**

| **OBS components** | **Property** | **Male** | | | **Female** | | |
| --- | --- | --- | --- | --- | --- | --- | --- |
|  |  | 0 | 1 | 2 | 0 | 1 | 2 |
| Dietary fiber (g/d) | A | <12.85 | 12.85-19.15 | ≥19.15 | <13.17 | 13.17-17.88 | ≥17.88 |
| Carotene (RE/d) | A | <52.91 | 52.91-177.72 | ≥177.72 | <61.46 | 61.46-210.98 | ≥210.98 |
| Riboflavin (mg/d) | A | <1.90 | 1.90-2.59 | ≥2.59 | <1.78 | 1.78-2.36 | ≥2.36 |
| Niacin (mg/d) | A | <22.47 | 22.47-29.87 | ≥29.87 | <20.17 | 20.17-26.08 | ≥26.08 |
| Vitamin B6 (mg/d) | A | <1.72 | 1.72-2.42 | ≥2.42 | <1.59 | 1.59-2.08 | ≥2.08 |
| Total folate (mcg/d) | A | <331.18 | 331.18-482.14 | ≥482.14 | <319.27 | 319.27-417.00 | ≥417.00 |
| Vitamin B12 (mcg/d) | A | <3.83 | 3.83-6.60 | ≥6.60 | <3.60 | 3.60-5.76 | ≥5.76 |
| Vitamin C (mg/d) | A | <46.26 | 46.26-107.11 | ≥107.11 | <53.14 | 53.14-106.54 | ≥106.54 |
| Vitamin E (ATE) (mg/d) | A | <5.62 | 5.62-8.07 | ≥8.07 | <5.66 | 5.66-7.76 | ≥7.76 |
| Calcium (mg/d) | A | <721.18 | 721.18-1047.02 | ≥1047.02 | <745.19 | 745.19-1037.90 | ≥1037.90 |
| Magnesium (mg/d) | A | <256.72 | 256.72-336.65 | ≥336.65 | <250.45 | 250.45-321.16 | ≥321.16 |
| Zinc (mg/d) | A | <10.42 | 10.42-14.02 | ≥14.02 | <9.87 | 9.87-12.63 | ≥12.63 |
| Copper (mg/d) | A | <1.16 | 1.16-1.50 | ≥1.50 | <1.13 | 1.13-1.44 | ≥1.44 |
| Selenium (mcg/d) | A | <99.95 | 99.95-128.63 | ≥128.63 | <93.69 | 93.69-114.75 | ≥114.75 |
| Total fat (g/d) | P | ≥91.48 | 72.36-91.48 | <72.36 | ≥85.49 | 70.47-85.49 | <70.47 |
| Iron (mg/d) | P | ≥18.60 | 13.49-18.60 | <13.49 | ≥16.40 | 12.85-16.40 | <12.85 |
| Physical activity(MET-minute/week) | A | ＜252 | 252-822.11 | ≥822.11 | ＜252 | 252-756 | ≥756 |
| Alcohol (g/d) | P | ＞28 | 0-28 | 0 | ＞14 | 0-14 | 0 |
| BMI (kg/m^2^) | P | ≥30.0 | 25.0-29.9 | ＜25.0 | ≥30.0 | 25.0-29.9 | ＜25.0 |
| Cotinine (ng/mL) | P | ≥1.25 | 0.03-1.25 | ＜0.03 | ≥0.11 | 0.02-0.11 | ＜0.02 |

OBS: oxidative balance score; A: antioxidant; P: prooxidant; RE: retinol equivalent; ATE: alpha-tocopherol equivalent; MET: metabolic equivalent; BMI: body mass index

**Table S8. Sensitivity Analysis 3: Associations Between Total, Dietary, and Lifestyle OBS and Allergic Rhinitis After Energy Adjustment**

| Characteristic | Sensitivity analysis 3 | |
| --- | --- | --- |
|  | OR(95% CI) | *P* |
| Total OBS | 1.02 (1.00, 1.04) | 0.024 |
| Total OBS (per SD) | 1.15 (1.02, 1.29) | 0.024 |
| Total OBS quartile |  |  |
| Q1 | Ref. | - |
| Q2 | 1.28 (0.93, 1.77) | 0.130 |
| Q3 | 1.28 (0.92, 1.78) | 0.140 |
| Q4 | 1.53 (1.10, 2.14) | 0.013 |
| *P* for trend |  | 0.018 |
| Dietary OBS | 1.02 (1.00, 1.04) | 0.019 |
| Dietary OBS (per SD) | 1.15 (1.02, 1.30) | 0.019 |
| Dietary OBS quartile |  |  |
| Q1 | Ref. | - |
| Q2 | 1.44 (1.00, 2.08) | 0.051 |
| Q3 | 1.53 (1.06, 2.22) | 0.025 |
| Q4 | 1.85 (1.29, 2.69) | 0.001 |
| *P* for trend |  | <0.001 |
| Lifestyle OBS | 1.01 (0.93, 1.08) | 0.900 |
| Lifestyle OBS (per SD) | 1.01 (0.90, 1.14) | 0.900 |
| Lifestyle OBS quartile |  |  |
| Q1 | Ref. | - |
| Q2 | 0.89 (0.65, 1.22) | 0.500 |
| Q3 | 0.98 (0.71, 1.35) | 0.900 |
| Q4 | 0.96 (0.69, 1.33) | 0.800 |
| *P* for trend |  | 0.945 |

Sensitivity analysis 3: Models were adjusted for age, gender, race/ethnicity, education level, PIR, serum CRP, smoking status, hypertension, diabetes, CVD, and dietary supplement use.

**Table S9. Sensitivity Analysis 4: Associations Between Total and Dietary OBS and Allergic Rhinitis Under Alternative Definitions of Allergic Rhinitis**

| Characteristic | sIgE ≥ 0.35 kU/L | | sIgE ≥ 0.70 kU/L | |
| --- | --- | --- | --- | --- |
|  | OR(95% CI) | *P* | OR(95% CI) | *P* |
| Total OBS | 1.02 (1.01, 1.04) | 0.010 | 1.03 (1.01, 1.05) | 0.002 |
| Total OBS (per SD) | 1.16 (1.04, 1.30) | 0.010 | 1.21 (1.07, 1.36) | 0.002 |
| Total OBS quartile |  |  |  |  |
| Q1 | Ref. | - | Ref. | - |
| Q2 | 1.22 (0.89, 1.66) | 0.200 | 1.18 (0.85, 1.64) | 0.300 |
| Q3 | 1.41 (1.03, 1.93) | 0.030 | 1.56 (1.12, 2.17) | 0.008 |
| Q4 | 1.61 (1.17, 2.22) | 0.003 | 1.78 (1.28, 2.50) | <0.001 |
| *P* for trend |  | 0.002 |  | <0.001 |
| Dietary OBS | 1.02 (1.01, 1.04) | 0.012 | 1.03 (1.01, 1.05) | 0.002 |
| Dietary OBS (per SD) | 1.16 (1.03, 1.29) | 0.012 | 1.20 (1.07, 1.35) | 0.002 |
| Dietary OBS quartile |  |  |  |  |
| Q1 | Ref. | - |  |  |
| Q2 | 1.27 (0.93, 1.72) | 0.130 | 1.15 (0.83, 1.60) | 0.400 |
| Q3 | 1.34 (0.99, 1.83) | 0.062 | 1.35 (0.97, 1.87) | 0.074 |
| Q4 | 1.46 (1.07, 2.00) | 0.018 | 1.59 (1.14, 2.20) | 0.006 |
| *P* for trend |  | 0.018 |  | 0.003 |

Sensitivity analysis 4: Models were adjusted for age, gender, race/ethnicity, education level, PIR, serum CRP, smoking status, hypertension, diabetes, CVD, and dietary supplement use.
